# Target capture data resolve recalcitrant relationships in the coffee family (Rubioideae, Rubiaceae)

**DOI:** 10.3389/fpls.2022.967456

**Published:** 2022-09-08

**Authors:** Olle Thureborn, Sylvain G. Razafimandimbison, Niklas Wikström, Catarina Rydin

**Affiliations:** ^1^Department of Ecology, Environment and Plant Sciences, Stockholm University, Stockholm, Sweden; ^2^Department of Botany, Swedish Museum of Natural History, Stockholm, Sweden; ^3^Bergius Foundation, Royal Swedish Academy of Sciences, Stockholm, Sweden

**Keywords:** Angiosperms353, incomplete lineage sorting, non-coding DNA, nuclear phylogeny, phylogenomics, Rubiaceae, Rubioideae, target capture

## Abstract

Subfamily Rubioideae is the largest of the main lineages in the coffee family (Rubiaceae), with over 8,000 species and 29 tribes. Phylogenetic relationships among tribes and other major clades within this group of plants are still only partly resolved despite considerable efforts. While previous studies have mainly utilized data from the organellar genomes and nuclear ribosomal DNA, we here use a large number of low-copy nuclear genes obtained via a target capture approach to infer phylogenetic relationships within Rubioideae. We included 101 Rubioideae species representing all but two (the monogeneric tribes Foonchewieae and Aitchinsonieae) of the currently recognized tribes, and all but one non-monogeneric tribe were represented by more than one genus. Using data from the 353 genes targeted with the universal Angiosperms353 probe set we investigated the impact of data type, analytical approach, and potential paralogs on phylogenetic reconstruction. We inferred a robust phylogenetic hypothesis of Rubioideae with the vast majority (or all) nodes being highly supported across all analyses and datasets and few incongruences between the inferred topologies. The results were similar to those of previous studies but novel relationships were also identified. We found that supercontigs [coding sequence (CDS) + non-coding sequence] clearly outperformed CDS data in levels of support and gene tree congruence. The full datasets (353 genes) outperformed the datasets with potentially paralogous genes removed (186 genes) in levels of support but increased gene tree incongruence slightly. The pattern of gene tree conflict at short internal branches were often consistent with high levels of incomplete lineage sorting (ILS) due to rapid speciation in the group. While concatenation- and coalescence-based trees mainly agreed, the observed phylogenetic discordance between the two approaches may be best explained by their differences in accounting for ILS. The use of target capture data greatly improved our confidence and understanding of the Rubioideae phylogeny, highlighted by the increased support for previously uncertain relationships and the increased possibility to explore sources of underlying phylogenetic discordance.

## Introduction

The subfamily Rubioideae, the largest of the major lineages of the species-rich and morphologically diverse coffee family (the Rubiaceae), includes over 8,000 species (Wikström et al., 2020). The members of the subfamily are characterized as herbs or shrubs (rarely trees) with tissues containing raphides (calcium oxalate crystals), valvate corolla aestivation, indumentum of septate hairs and heterostylous flowers (e.g., Robbrecht, 1988; Bremer and Manen, 2000; Robbrecht and Manen, 2006; Bremer and Eriksson, 2009). As for the remaining family, most species are found in tropical and subtropical regions around the world, however, several species of the tribes Anthospermeae, Putorieae, Rubieae, and Theligoneae are distributed in temperate regions. The wind-pollinated flowers in the tribes Anthospermeae and Theligoneae are also an unusual trait, relative to other Rubiaceae, found in this subfamily. The four aforementioned temperate tribes belong to one of the major clades within the subfamily, the cosmopolitan and mainly herbaceous Spermacoceae alliance, which contain over 3,000 species. Together the tribes Spermacoceae and Rubieae make up the bulk of species with more than 1,300 and 900 species, respectively (Wikström et al., 2020). The other major informal group of Rubioideae, the pan-tropical and mainly woody Psychotrieae alliance, also contains over 3,000 species, of which most belong to the tribes Palicoureeae and Psychotrieae, much due to the large genera *Psychotria* and *Palicourea*, with about 1,600 and 800 species, respectively (Razafimandimbison et al., 2008, 2014; Davis et al., 2009).

In total, Wikström et al. (2020) recognized 27 tribes in the subfamily Rubioideae in their summary, based on previous molecular phylogenetic studies. Recently two additional monospecific tribes have been described; the tribe Seychelleeae, which is sister to the tribe Colletoecemateae (Razafimandimbison et al., 2020), and the tribe Aitchinsonieae, which is placed in the Putorieae-Rubieae-Theligoneae clade (also referred to as the Rubieae complex, Bordbar et al., 2021). The Rubioideae thus include the two major groups the Psychotrieae and the Spermacoceae alliances, and seven additional tribes: Colletoecemateae, Seychelleeae, Urophylleae, Ophiorrhizeae, Lasiantheae, Perameae, and Coussareeae. The members of the Psychotrieae alliance are classified in nine tribes: Craterispermeae, Gaertnereae, Mitchelleae, Morindeae, Palicoureeae, Prismatomerideae, Psychotrieae, Schizocoleeae, and Schradereae. In the Spermacoceae alliance, 13 tribes are recognized: Aitchinsonieae, Argostemmateae, Anthospermeae, Cyanoneuroneae, Danaideae, Dunnieae, Foonchewieae, Knoxieae, Paederieae, Putorieae, Rubieae, Spermacoceae, and Theligoneae.

Until recent years, phylogenetic studies in the Rubioideae have mainly relied on information from selected plastid markers (e.g., *atpB-rbcL, rbcL, rps16, trnT-trnL-trnF, ndhF*) (Andersson and Rova, 1999; Bremer and Manen, 2000; Piesschaert et al., 2000; Robbrecht and Manen, 2006; Rydin et al., 2008; Bremer and Eriksson, 2009; Wikström et al., 2015; Janssens et al., 2016) or plastid markers combined with a few nuclear ribosomal regions (e.g., nrITS and/or nrETS) (Razafimandimbison et al., 2008, 2014; Antonelli et al., 2009; Rydin et al., 2009b; Razafimandimbison and Rydin, 2019). Such studies laid the foundation of the phylogenetic understanding within Rubioideae and the rest of the family. Recently, Rydin et al. (2017) and Wikström et al. (2020) used organellar genome scale datasets to reconstruct the phylogeny of the Rubiaceae family. Wikström et al. (2020) also analyzed nuclear ribosomal cistron data. Their results were mostly well supported and corroborated the overall picture of intertribal-relationships within Rubioideae, although high support values were not always achieved. Furthermore, results from the three different genomic compartments were not fully consistent (Rydin et al., 2017; Wikström et al., 2020). For example, deep-branching relationships within Rubioideae showed well supported yet conflicting tree topologies with either Ophiorrhizeae, a clade comprising Colletoecemateae and Urophylleae, or a clade comprising Colletoecemateae as sister to an Ophiorrhizeae + Urophylleae clade, resolved as sister group to the remaining subfamily. Another example of supported conflict was revealed by analysis of nuclear ribosomal data, which placed Coussareeae as sister to the Spermacoceae alliance, challenging the well documented sister-relationship between the Spermacoceae and Psychotrieae alliances in a number of previous studies (e.g., Bremer and Manen, 2000; Razafimandimbison et al., 2008; Rydin et al., 2009b). Relationships within the Psychotrieae and Spermacoceae alliances also differed between analyses of the different compartments, including deep splits within the Spermacoceae alliance, relationships among tribes of the Rubieae complex and the position of Gaertnereae in the Psychotrieae alliance. Antonelli et al. (2021) examined the higher-level relationships in the entire Gentianales using target capture data, and while results were mostly consistent with those of previous studies, some surprising relationships were retrieved among their results. For instance, the sister relationship between Argostemmateae and the remaining tribes of the Spermacoceae alliance in their coalescent tree based on nuclear data, and the placement of Cyanoneuroneae nested within Psychotrieae alliance based on plastid data (Antonelli et al., 2021).

However, these family- and order-wide phylogenies have as a rule included only one representative taxon per sampled tribe and some key taxa have been unsampled. Furthermore, analysis of an organellar genome is generally considered to represent a single gene-tree within the species phylogeny (Gitzendanner et al., 2018; Doyle, 2022) and can thus fail to reflect the correct species tree due to processes such as incomplete lineage sorting (ILS) and hybridization (Nicholls et al., 2015; Wolf et al., 2018). Sampling a large number of presumably independently evolving genetic loci can avoid such problems and may even be necessary to infer the correct species tree (Degnan and Rosenberg, 2009; Nicholls et al., 2015; Ruane et al., 2015).

Targeted sequence capture uses short (often RNA) probes that are designed for the group of study to selectively capture target DNA regions from sequencing libraries and has emerged as a standard method for generating genome-scale nuclear multi-gene datasets for species tree inference in several plant groups (Johnson et al., 2019; Hale et al., 2020). The relative cost effectiveness and the fact that it works well also with degraded DNA, which is common among extractions of herbarium specimens, are some benefits of this approach (McKain et al., 2018; Johnson et al., 2019). The probe set used may be specifically designed for the group of study (e.g., Vatanparast et al., 2018; Sanderson et al., 2020) or designed to be universally applicable across larger groups such as the Angiosperms353 probe kit (Johnson et al., 2019). The large amount and heterogeneity of the data generated for phylogenomic studies do, however, not come without challenges. Factors such as poorly resolved gene trees due to low phylogenetic signal (Zhang et al., 2018), different types of data (Braun and Kimball, 2021), different data filtering strategies (Molloy and Warnow, 2018), and different underlying assumptions of phylogenetic inference methods such as concatenation- and coalescent-based methods (Roch and Steel, 2015) may all potentially affect accuracy of species tree inference.

Here, we attempt to resolve the phylogeny of the subfamily Rubioideae using large amounts of target capture data from the nuclear genome, and a much denser sampling of taxa, including several representatives of nearly all tribes of the subfamily, compared to previous work. We examine the impact of data type [coding sequence (CDS) and CDS + non-coding sequence], analytical approach (coalescence and concatenation), and potential paralogs (inclusion/exclusion of putative paralogous genes) on phylogenetic reconstruction. Our main aim is to improve the understanding of relationships within Rubioideae, mainly among tribes but also within tribes.

## Materials and methods

### Taxon sampling

One hundred and one Rubioideae species were selected to obtain a good representation of the subfamily. These species included representatives from all but two (the monogeneric tribes Foonchewieae and Aitchinsonieae) of the currently recognized tribes, and all but one non-monogeneric tribe was represented by more than one genus. For outgroup sampling we included twenty species to represent the major lineages of the remaining Rubiaceae, including representatives from the two other subfamilies and the two unplaced tribes Coptosapelteae and Luculieae. Three outgroup species from the Gentianales families Gentianaceae, Loganiaceae, and Apocynaceae were also selected. For 93 species, material was selected from vegetative tissue material (either silica dried material from field collections or from herbarium specimens) or from DNA aliquots already available from previous work. We also downloaded raw sequence data from the European Nucleotide Archive for 31 species available via the Plant and Fungal Tree of Life (PAFTOL) Research Program (Baker et al., 2022). Species and voucher information for all included taxa is provided in Supplementary Table 1.

### Library preparation and target capture

DNA was extracted using a cetyl trimethylammonium bromide method (Doyle and Doyle, 1987). The plant tissue was pulverized using a TissueLyser LT (Qiagen, Hilden, Germany). Some samples were additionally cleaned with AMPure XP beads (Beckman Coulter, Indianapolis, IN, United States) or with a QIAquick polymerase chain reaction (PCR) kit (Qiagen, Hilden, Germany) according to the instructions provided by the manufacturers. DNA degradation was assessed by agarose gel (1%) electrophoresis and quantified on a Qubit 3 Flourometer (Thermo Fisher Scientific, Waltham, MA, United States) using the Qubit dsDNA HS kit. Samples with a large fraction of DNA fragments above 350 bp were placed in 96 microTUBE Plate wells and fragmented on a Covaris E220 Focused-ultrasonicator (Covaris, Woburn, MA, United States) using the program for a target insert size of 350 bp at Science for Life Laboratory (Solna, Sweden).

Libraries were prepared using a modified version of the Meyer and Kircher (2010) protocol. Briefly, the major steps of library preparation consisted of blunt-end repair, adapter ligation and adapter fill-in, followed by four separate index PCRs. End repair was performed in 40 μl reactions with 20 μl of DNA extract. AMPure bead cleanups after blunt-end repair and adapter ligation were performed using ratios of 0.9–1.8:1 AMPure to reaction volume. Adapter concentration in the ligation reaction was reduced to 0.25 μM of each adapter, and the cleanup step after adapter fill-in was substituted with heat inactivation of the Bst polymerase at 80°C for 20 min following Kircher et al. (2012).

Each adapter-ligated library was then amplified with P5 and P7 dual-indexing primers in four separate PCR reactions to reduce amplification bias. One initial 12 cycle PCR per library was performed and the PCR products were loaded on a 1% agarose gel to verify amplification success and to determine an appropriate number of cycles for the remaining PCRs. Each 25 μl reaction contained 7 μl DNA library template and the following final concentrations: 1 × PCR Gold buffer, 2.5 mM MgCl2, 0.25 mM of each dNTP, 200 nM of each primer and 5 U AmpliTaq Gold. Reactions were subjected to the following thermocycling conditions: 94°C 12 min; 6–14 cycles of 94°C for 30 s, 60°C for 30 s, 72°C for 45 s; and a final extension of 72°C for 10 min. Individual PCR products for each sample were then pooled and cleaned using AMPure XP beads using ratios of 0.85-1:1 AMPure to reaction volume. The specific ratio used varied depending on DNA degradation, concentration and amount of unwanted short fragments (e.g., adapter-dimers) of the samples. The cleaned libraries were quantified using the Qubit dsDNA HS kit on a Qubit 3 Flourometer and fragment size distribution inspected with a high-sensitivity DNAchip on a Bioanalyzer 2100 (Agilent, Santa Clara, CA, United States).

Libraries of similar size were combined into 6-plex or 8-plex pools resulting in approximately equimolar 600 and 800 ng pools, respectively. Before pooling, apart from fragment size distribution, other factors, such as tissue source, number of PCR cycles during library preparation, age and library concentration were also considered. The pools were concentrated using either a miVac (Genevac, Ipswich, United Kingdom) or SpeedVac (Thermo Fisher Scientific, Waltham, MA, United States) at approximately 43°C. The pools were then enriched with the myBaits Expert Predesigned Panel (Arbor Biosciences, Ann Arbor, MI, United States) Angiosperms353 v1 (Catalog #308196; Johnson et al., 2019) following the manufacturer’s protocol (v4).^1^ Hybridization was carried out at 62°C for 24 or 36 h. Enriched products were amplified with KAPA HiFi (2×) HotStart ReadyMix PCR Kit (Roche, Basel, Switzerland) for 13–14 cycles with IS5_reamp. P5 and IS6_reamp.P7 primers (Meyer and Kircher, 2010) and subsequently cleaned using a 0.9:1 AMPure to reaction volume ratio. The hybridized and cleaned pools were quantified using the Qubit dsDNA HS kit and fragment size distribution inspected with a high-sensitivity DNAchip on a Bioanalyzer 2100. Finally, the enriched library pools were multiplexed at equimolar concentrations and sequenced on a NextSeq 500 using “Mid-Output” chemistry or NovaSeq 6000 using “NovaSeqXp” workflow in “S4” mode flowcell (Illumina, San Diego, CA, United States) with 151 bp paired-end reads at Science for Life Laboratory (Solna, Sweden).

### Data pre-processing

The Bcl to FastQ conversion was performed using bcl2fastq_v2.20.0.422 from the CASAVA software suite +, at Science for Life Laboratory (Solna, Sweden). The quality scale used was Sanger/phred33/Illumina 1.8. Further preprocessing of the obtained 151 bp paired-end reads was performed using utilities in the BBTools suite (BBTools, 2022). Dedupe or alternatively Clumpify was used to remove duplicate reads. BBduk was used to trim adapters, trim low-quality bases (Q < 20) and remove reads shorter than 36 bp. Dedupe and BBduk were used from within Geneious 11.1.5 (Kearse et al., 2012).

### Gene assemblies

HybPiper v1.3.1 (Johnson et al., 2016) was used to assemble sequences for each gene. With the aim to increase gene recovery (gene length and number) the default target file for the Angiosperms353 kit was expanded by adding sequences of the Gentianales samples included in the mega353 target file produced by McLay et al. (2021) and the 348 sequences from the annotated *Coffea canephora* genome available via The Kew Tree of Life Explorer (Baker et al., 2022). The reads of library replicates from the same sample were combined before assembly. Read mapping was conducted using BWA v0.7.17 (Li and Durbin, 2009) and the coverage cut-off option was kept at the default value of eight for the SPAdes v3.15.2 (Bankevich et al., 2012) contig assembly. In addition to the default HybPiper coding sequence (CDS) output extracted with exonerate v2.2 (Slater and Birney, 2005) the optional HybPiper intronerate.py script was run to also extract so called supercontig sequences, which contain both CDS and non-coding flanking sequence. Recovery statistics were generated using the two HybPiper scripts get_seq_lengths.py and hybpiper_stats.py. The HybPiper script paralog_investigator.py was run to identify genes with paralog warnings. A HybPiper paralog warning is generated when HybPiper assembles multiple contigs covering more than 85% of the target length. In such a case HybPiper selects the sequence with highest sequencing coverage. If the copies have similar coverage, the copy with highest percent identity to the target sequence is chosen.

### Alignment, dataset generation and phylogenetic analysis

The CDS and supercontig outputs for each target gene were aligned with MAFFT v7.467 (Katoh and Standley, 2013) with the L-INS-I algorithm and the additional –adjust direction flag. CDS alignments were aligned as amino acids and backtranslated using PAL2NAL v14 (Suyama et al., 2006). BMGE v1.12 (Criscuolo and Gribaldo, 2010) was used to trim sites with more than 90% gaps. The trimmed alignments were then concatenated using AMAS v1.0 (Borowiec, 2016), and Spruceup v2020.2.19 (Borowiec, 2019) was used to detect and trim outlier sequence windows from individual samples using the Jukes-Cantor-corrected distance method, a window size of 20 bp, an overlap size of 15 bp, a lognormal distribution and a cutoff value of 0.99. AMAS was then used to split the concatenated alignment into single-locus alignments and again trimmed with BMGE to remove sites with more than 90% gaps. The resulting alignments were used for phylogenetic inference. Alignment length, number and proportion of parsimony informative sites (PIS) and other alignment statistics were obtained using AMAS.

A total of four datasets were created. For each data type we created a dataset comprising the full set of genes (i.e., the direct HybPiper output), which we refer to as the full CDS dataset and full supercontig dataset. We also created a putative one-to-one ortholog dataset for each data type, which we refer to as the paralog-filtered CDS dataset and the paralog-filtered supercontig dataset. The two paralog-filtered datasets were created by conservatively removing any gene with at least one paralog warning from the respective full set of genes. The datasets were analyzed using a coalescent approach and a concatenation approach.

We used IQ-TREE 2 v2.0.3 (Minh et al., 2020) to infer a gene tree for each single gene alignment under the GTR + G model with support assessed with 1,000 ultrafast bootstrap replicates (Hoang et al., 2018). Following gene tree estimation, we collapsed nodes with less than 20% support using Newick Utilities v1.6 (Junier and Zdobnov, 2010) as this can help improve gene tree accuracy (Zhang et al., 2018). We then used the collapsed gene trees for species tree inference with a coalescent-based approach, using the quartet-based summary method ASTRAL III v5.7.8 (Zhang et al., 2018), which accounts for gene tree discordance due to ILS. Node support was assessed by local posterior probability (LPP; Sayyari and Mirarab, 2016). We also performed the polytomy test implemented in ASTRAL, which uses quartet gene tree frequencies to evaluate whether polytomies could be rejected at short branches (Sayyari and Mirarab, 2018). The normalized quartet score (NQS), which reflects the percentage of the gene tree quartets included in the species tree and part of the ASTRAL output, was used to assess the level of gene tree discordance for the respective datasets. To further examine gene tree discordance ASTRAL trees were annotated with quartet frequencies for alternative topologies using the –t 8 option in ASTRAL-III.

For each of the four datasets we also concatenated the single gene alignments to infer phylogenies in a concatenation framework. The concatenated matrices were analyzed using IQ-TREE 2 using a partitioned model (Chernomor et al., 2016), with each gene treated as a separate partition with a GTR + G model specified for each partition and allowing the possibility of separate rates among partitions. To assess branch support, ultrafast bootstrap supports (BS) were calculated based on 1,000 replicates.

Treeio (Wang et al., 2020) and ape (Paradis and Schliep, 2019) R packages (R Core Team, 2022) were used to plot the trees followed by editing in Inkscape v1.1.2 (Inkscape Project, 2022).

## Results

### Sequencing and assembly statistics

Sequencing and data filtration results can be found in Supplementary Table 1. Across all newly generated libraries the number of deduplicated and trimmed reads had a mean of 14,535,279. Across all libraries (i.e., including also the 31 PAFTOL samples downloaded from ENA) the number of deduplicated and trimmed reads had a mean of 11,653,388. The average library had 23% duplicate reads removed.

Assembly results are provided in Supplementary Table 2. At least a fraction of each of the 353 targeted genes were recovered in at least five taxa. Across the newly sequenced samples, the average sample had 336, 312, and 263 genes with sequences at least 25, 50, and 75% of the average target length, respectively, and a total gene length of 245,218 bp. Across all samples the average sample had 323, 291, and 237 genes with sequences at least 25, 50, and 75% of the average target length, respectively, and a total gene length of 228,644 bp. In addition to the targeted coding regions, large amounts of non-targeted sequence data were recovered. The average total length of recovered supercontig (coding sequence and non-coding flanking sequence) data was 710,450 and 661,303 bp for the newly sequenced samples and all samples, respectively. Across the full taxon sample, HybPiper gave paralog warnings for at least one sample in 167 of 353 genes. On average, samples had nine paralog warnings.

### Dataset characteristics

The main characteristics of the four assembled datasets are summarized in Table 1 and full statistics for each single locus alignment are provided in Supplementary Table 3. Across the 353 loci the average final alignment had a taxon coverage of 94% (117/124 species), and a length of 880 and 2,989 bp for the CDS and supercontig datasets, respectively. The total concatenated length of the full CDS dataset was 310,806 bp and the full supercontig dataset was 1,055,164 bp. The exclusion of the putatively paralogous genes (i.e., the genes flagged with paralog warnings by HybPiper) resulted in 186 alignments each for the paralog-filtered datasets with a total concatenated length of 181,088 and 632,932 bp for the CDS and supercontig datasets, respectively. On average, supercontig alignments contained over five times more PIS than CDS alignments.

**TABLE 1:** Characteristics of assembled datasets used for phylogenetic inference.

Dataset	# Of loci	Concatenated length	# Of PIS (%)	Average taxon coverage (%)	Average alignment length	Average PIS per locus	Average percentage PIS per locus
Full supercontig	353	1,055,164	876,813 (83.1%)	117/124 (94.4%)	2,989	2,484	82.7
Full CDS	353	310,806	169,772 (54.6%)	117/124 (94.4%)	880	481	53.6
Paralog-filtered supercontig	186	632,932	526,877 (83.2%)	115/124 (92.7%)	3,403	2,833	82.9
Paralog-filtered CDS	186	181,088	99,394 (54.9%)	115/124 (92.7%)	974	534	53.7

PIS, parsimony informative sites.

### Comparison of data types and inclusion/exclusion of potential paralogous genes

The performance of the four datasets on branch support, gene tree discordance (NQS values) and ability to reject polytomies are summarized in Table 2. Across both gene sets (i.e., inclusion/exclusion of putatively paralogous genes) and analytical approaches, the addition of non-coding sequences increased the average branch support, number of branches where a polytomy could be rejected, number of highly supported nodes, and gene tree concordance (i.e., higher NQS values). For the coalescence-based analyses of the full and paralog-filtered datasets there were nine (ingroup = seven) and 12 (ingroup = nine) more strongly supported nodes when using supercontigs instead of CDS alone, respectively. For the concatenated analyses of the full and paralog-filtered datasets there were 10 (ingroup = seven) and six (ingroup = five) more strongly supported nodes when using supercontigs instead of CDS alone, respectively. The number of branches where a polytomy could be rejected using the polytomy test in ASTRAL in the analyses of the full and paralog-filtered datasets was also higher when supercontigs were used instead of CDS alone, increasing with six (ingroup = four) and nine (ingroup = five) branches, respectively. Across both gene sets, supercontigs increased average BS support with 2.1% for the full and paralog-filtered datasets. Across both gene sets, supercontigs increased average LPP support with 1.9 and 2.8% for the full and paralog-filtered datasets, respectively. Across both gene sets the addition of flanking regions resulted in higher NQS values, increasing with 0.050 and 0.057 for the full and paralog-filtered datasets, respectively.

**TABLE 2:** Phylogenetic inference performance of the assembled datasets for attributes under consideration.

Phylogenetic inference approach						
	Coalescence (ASTRAL)				Concatenation (IQ-TREE)	
Dataset	Normalized quartet score	# Of branches below < 95% ingroup| global	# Of branches for which a polytomy could not be rejected. ingroup| global	Average LPP	# Of branches below < 95% ingroup| global	Average BS
Full supercontig	0.930	6| 8	4| 5	0.983	0| 0	99.9
Full CDS	0.880	13| 17	8| 11	0.964	7| 10	97.8
Paralog-filtered supercontig	0.939	8| 11	9| 10	0.973	1| 4	99.6
Paralog-filtered CDS	0.882	17| 23	14| 19	0.945	6| 10	97.5

Across both data types and analytical approaches, the exclusion of genes with putative paralogs reduced the average branch support, number of branches where a polytomy could be rejected, and number of highly supported nodes, except for the concatenated analyses of CDS data where the exclusion of putatively paralogous genes resulted in one more well-supported ingroup branch. Excluding putatively paralogous genes from the supercontig data, the number of strongly supported nodes was reduced by four (ingroup = one) for the concatenation-based analysis. Excluding putatively paralogous genes from the supercontig and CDS data, the number of strongly supported nodes was reduced by three (ingroup = two) and six (ingroup = four) nodes for the coalescence-based analyses, respectively. Excluding putatively paralogous genes from supercontig and CDS data, the number of branches where a polytomy could be rejected decreased by five (ingroup = five) and eight (ingroup = six) branches, respectively. Across both data types, excluding putatively paralogous genes decreased average BS support by 0.3% for the full and paralog-filtered datasets. Across both gene sets, excluding putatively paralogous genes decreased average LPP support by 1 and 1.9% for the supercontig and CDS datasets, respectively. However, across both data types the removal of putatively paralogous genes resulted in slightly higher NQS values, with an increase of 0.002 and 0.009 for the CDS and supercontig datasets, respectively.

### Phylogenetic results

**FIGURE 1:**
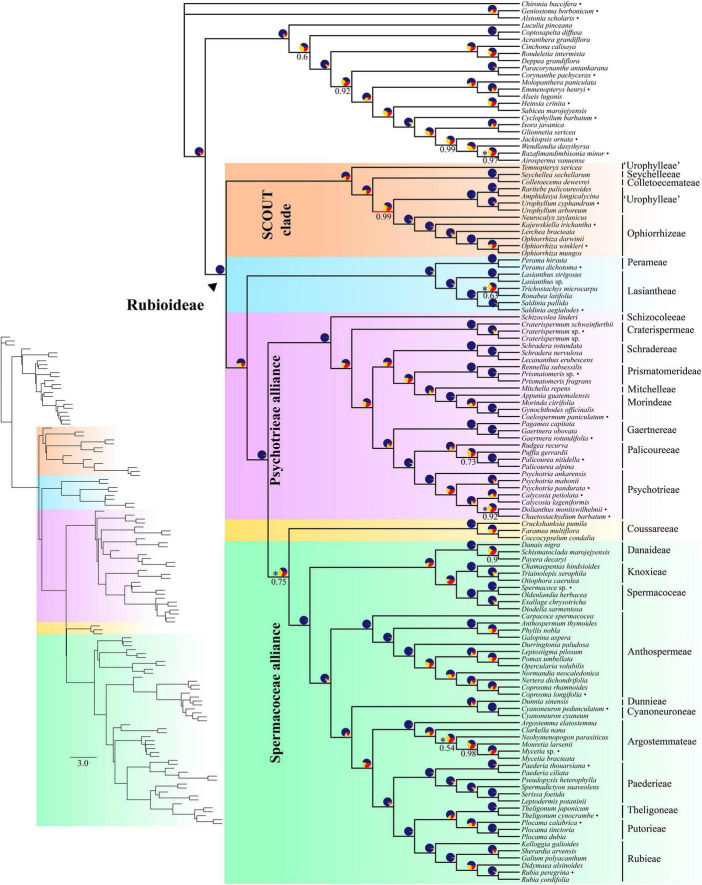
Coalescent-based species tree estimated using ASTRAL on the full supercontig dataset. Numbers below branches denote local posterior probability (LPP) support values. Only support values smaller than 100% are shown. Pie charts show relative frequencies of the three quartet topologies around the branch (blue = congruent with species tree, yellow = first alternative topology, red = second alternative topology). Asterisks next to pie charts indicate failure to reject the hypothesis that the branch is a polytomy. Bullets after species names indicate samples downloaded from ENA. Inset shows branch lengths in coalescent units.

**FIGURE 2:**
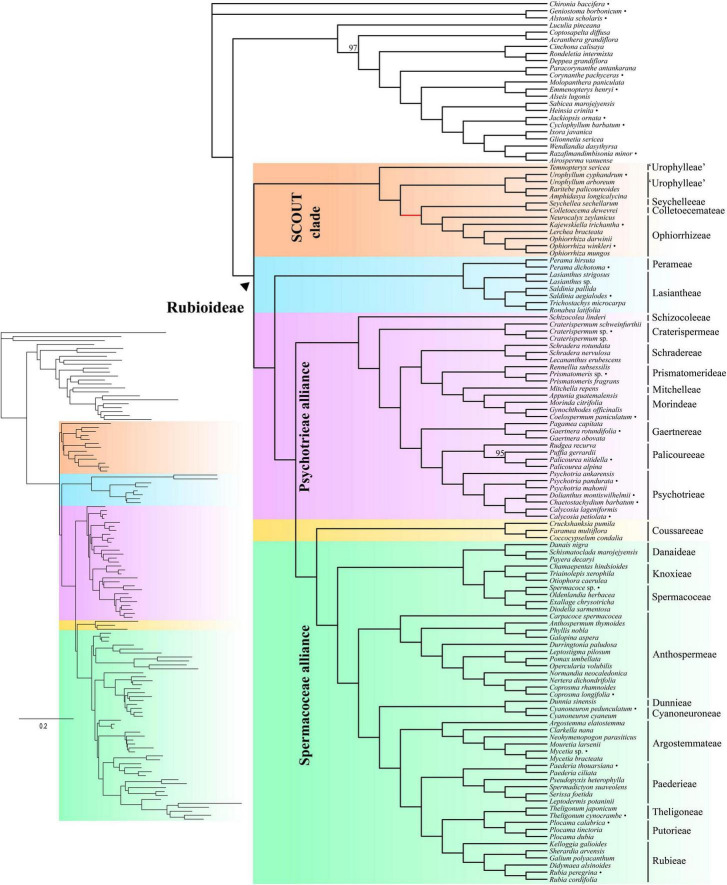
Concatenation-based tree estimated using IQ-TREE on the full supercontig dataset. Numbers above branches denote ultrafast bootstrap (BS) support values. Only support values smaller than 100% are shown. Bullets after species names indicate samples downloaded from ENA. Inset shows branch lengths in number of substitutions per site.

The inferred species tree topologies were highly similar regardless of method (coalescence- or concatenation-based), data type (CDS or supercontigs) and inclusion/exclusion of potentially paralogous genes (Figures 1, 2 and Supplementary Figures 1–6). The few topological conflicts were often not well supported (i.e., were supported by less than 95%). Overall, both the addition of flanking regions and inclusion of all genes increased statistical support and the power to reject polytomies. Therefore, we in the following, focus on the results obtained from the analyses of the full supercontig dataset (Figures 1, 2).

#### Monophyly of Rubioideae, alliances, and tribes

Rubioideae, the Spermacoceae and Psychotrieae alliances, and all tribes except Urophylleae were highly supported as monophyletic (Figures 1, 2). Urophylleae as delimited by Smedmark et al. (2008) was never monophyletic in any of the inferred species trees. However, Urophylleae excluding *Temnopteryx* was always highly supported as monophyletic (Figures 1, 2), and this clade will hereafter be referred to as Urophylleae sensu stricto (s.s.).

#### Rubioideae backbone

Colletoecemateae, Ophiorrhizeae, Seychelleeae, Urophylleae s.s., and the genus *Temnopteryx* formed a clade (hereafter referred to as the SCOUT clade) sister to remaining Rubioideae, followed by a Lasiantheae + Perameae clade, the Psychotrieae alliance and a clade that joins the tribe Coussareeae and the Spermacoceae alliance (Figures 1, 2). All these relationships were, with one exception, strongly supported and polytomies were rejected. The exception was the sister relationship between Coussareeae and the Spermacoceae alliance, which was strongly supported (BS = 100) in the concatenated analysis (Figure 2) but had low support (LPP = 0.75) in the coalescence-based tree (Figure 1) and a polytomy could not be rejected.

#### SCOUT clade

Relationships within the SCOUT clade differed between analytical approaches. The coalescence-based tree resolved the genus *Temnopteryx* as sister to the remaining members, followed by a Seychelleeae + Colletoecemateae clade, and a Urophylleae s.s. + Ophiorrhizeae clade (Figure 1). The concatenation-based tree instead resolved Ophiorrhizeae as sister to the Seychelleeae + Colletoecemateae clade (Figure 2). Support for these sets of relationships was high for all nodes and polytomies were rejected.

#### Psychotrieae alliance

The tribe Schizocoleeae and Craterispermeae were successive sisters to the remaining Psychotrieae alliance; this last clade was in turn resolved in two sister lineages: A clade formed by Schradereae, Prismatomerideae, Morindeae and Mitchelleae and a clade uniting Gaertnereae and Psychotrieae + Palicoureeae (Figures 1, 2). Within the former clade, Schradereae and Prismatomerideae are successive sisters to Morindeae plus Mitchelleae (Figures 1, 2). Support for this set of relationships was high for all nodes and polytomies were rejected.

#### Spermacoceae alliance

In the Spermacoceae alliance a clade that joins Danaideae and Spermacoceae + Knoxieae was together sister to the remaining tribes, followed by Anthospermeae, a clade that joins Dunnieae + Cyanoneuroneae, Argostemmateae, Paederieae, and a clade that joins Theligoneae and Putorieae and Rubieae (Figures 1, 2). Support for this set of relationships was high for all nodes and polytomies were rejected.

## Discussion

The most comprehensive multigene phylogenetic analysis of Rubioideae yet published is presented here. The vast majority of nodes was strongly supported (≥ 95%) in both the coalescence-based and concatenation-based phylogenies (Figures 1, 2). We analyzed the data using a coalescent approach as well as a concatenation approach to phylogenetic inference of this group of plants, and we tested the inclusion/exclusion of putatively paralogous genes and the added information of non-targeted flanking regions in order to explore if relationships are reliant on a specific dataset or method. Leveraging substantial amounts of nuclear low-copy genetic data from a comprehensive taxon sample allowed us to infer a robust phylogenetic framework for the Rubioideae, potentially resolving and clarifying previously contentious relationships across the phylogeny of the group. For example, all inter-tribal relationships within the Spermacoceae and Psychotrieae alliances are robustly supported. Our study further supports the sister relationship between Coussareeae and the Spermacoceae alliance previously reported by Wikström et al. (2020) based on nuclear ribosomal cistron data. Within the Psychotrieae alliance, the Southeast Asian genus *Lecananthus* is nested in *Schradera*, more closely related to the Asian species *Schradera nervulosa* than either is to the neotropical *Schradera rotundata. Clarkella* is clearly included in the Argostemmateae, and *Pseudopyxis* in the Paederieae. The last three results are not unexpected considering morphological and geographic data. Furthermore, *Temnopteryx* is excluded from all currently described tribes of Rubiaceae; it is sister to remaining taxa in a well-supported SCOUT clade also comprising the tribes Seychelleeae, Colletoecemateae, Ophiorrhizeae and Urophylleae s.s., which together are supported as sister group to the remaining Rubioideae. Our study also shows that target capture data can resolve phylogenetic relationships with high confidence even in situations involving short branches, especially so when the combined information of coding and non-coding regions are used. Overall, our results indicate that ILS due to rapid diversification is likely one of the major underlying causes responsible for most of the phylogenetic incongruences at short branches in the Rubioideae phylogeny.

### Impact of potential paralogs, data type, and analytical method on phylogenetic inference

Inclusion of paralogous sequences can have important consequences for phylogenetic inference (Fitch, 1970; Yang and Smith, 2014). However, the topological results based on the full and paralog-filtered datasets mainly agree and statistical support increases when all genes are used. These factors suggest that (potential) paralogy did not change the topological results in any significant way, although the NQS values indicated slightly less gene tree discordance in the paralog-filtered data. This is in line with the results of Yan et al. (2021), which showed that ASTRAL and other coalescence-based methods are robust to species tree inference also in the presence of paralogs. Their study did, however, not include analyses of concatenated datasets, in which outlier genes have been shown to have extreme impact on topological results (Brown and Thomson, 2017). We used the target-capture data assembly HybPiper pipeline to assemble our datasets. This pipeline identifies paralogous copies and by default selects one copy based on sequencing coverage and percent identity to the target sequence. In other words, one copy per sample for each gene is selected and the approach is often applied to assemble target capture datasets (e.g., Antonelli et al., 2021; Clarkson et al., 2021; Maurin et al., 2021). However, this method may also flag genes with allelic variants rather than paralogs (Johnson et al., 2016) and may not uncover all paralogs (Zhou et al., 2022). Hence, both over- and underestimation of the number of detected putative paralogs is a possible outcome. Another common approach to deal with paralogs is to exclude entire genes that show evidence of paralogy, e.g., by removing putatively paralogous genes flagged by HybPiper (e.g., Larridon et al., 2020; Christe et al., 2021; Kuhnhäuser et al., 2021). Here, this approach resulted in a severe reduction of available sequence data left for species tree inference, which is common when many species are sampled (Emms and Kelly, 2018; Jones et al., 2019). This strict reliance on one-to-one orthologs led to an overall decrease in support and is likely to be an overly conservative approach in many phylogenetic contexts. Although (potential) paralogy did not seem to have any significant impact on the topological results presented in this paper, a more thorough analysis of paralogy may be worthwhile for future studies of subclades (e.g., genera) of Rubioideae. For example, identified paralogous copies could be used as additional loci (Gardner et al., 2021).

One advantage of targeted enrichment sequencing is that it facilitates assembly of non-targeted exon-flanking regions, including introns and sequence 5′ and 3′ to CDSs (Weitemier et al., 2014). Using the combined information of targeted CDS and non-targeted non-coding flanking sequence (supercontigs) improved overall statistical support as measured by number of highly supported nodes and average statistical support when compared to analyses of targeted CDS regions only. This finding is corroborated by other studies that have demonstrated increased statistical support for relationships by addition of flanking regions (e.g., Jones et al., 2019; Bagley et al., 2020; Gardner et al., 2021; Thomas et al., 2021). Addition of flanking regions also increased gene tree concordance and the power to reject polytomies with the polytomy test implemented in ASTRAL. Highly variable non-coding regions can be difficult to align but conserved flanking exons can help improve accuracy by anchoring the alignment (Gardner et al., 2021). Non-coding regions generally have higher evolutionary rates relative to CDS and should therefore contain more phylogenetic information, which may be necessary in order to resolve rapid speciation events (Chen et al., 2017). On the other hand, the higher variability (both in length and evolutionary rate) of non-coding regions may lead to higher degrees of noise. The overall higher statistical support we obtained using supercontig sequences and higher NQS values indicate that potential noise is overcome by the increased signal contained in these larger datasets.

It is notable, however, that there is one supported intertribal conflict between the paralog-filtered CDS and supercontig coalescence-based trees. While the analysis of the paralog-filtered supercontig data supported a Knoxieae + Danaideae clade (LPP = 0.96; Supplementary Figure 3), the paralog-filtered CDS data supported a Knoxieae + Spermacoceae clade (Supplementary Figure 5; LPP = 1). The latter relationship is highly supported in all other analyses in this study (including the concatenated analysis of the paralog-filtered dataset) and is also well established based on previous analyses of organellar and nuclear ribosomal DNA (Rydin et al., 2017; Wikström et al., 2020). Inspection of quartet frequencies shows that the two alternative quartet frequencies around the Knoxieae + Danaideae branch are not close (Supplementary Figure 1). This is contrary to the expectation of matching frequencies between the two alternative topologies if incongruence is due to ILS, indicating that sources of discordance other than ILS are involved, such as gene tree estimation error or gene flow (Degnan and Rosenberg, 2009; Leebens-Mack et al., 2019). The failure of the paralog-filtered dataset to resolve the Knoxieae + Spermacoceae relationship may be due to the much lower gene sampling in that dataset. However, the two alternative quartet frequencies around the Knoxieae + Spermacoceae branch in the full supercontig tree are also not close (Figure 1). Interestingly, the two alternative quartet frequencies around the Knoxieae + Spermacoceae branch in the two CDS trees (Supplementary Figures 3, 5) are similar and more indicative of ILS as the main source of discordance. A possible explanation for the patterns of quartet frequencies between analysis of CDS and supercontig data is that the highly variable non-coding regions of the supercontigs introduce gene tree estimation error due to noise in this part of the tree. Another possible explanation could be that introgression of DNA is biased toward non-coding regions following hybridization.

Gene tree heterogeneity is widespread in multigene datasets (Edwards et al., 2016). Potential biological reasons for gene tree incongruence include ILS, hybridization, and gene duplication and loss (Maddison, 1997). Of these, ILS, which is modeled by the multispecies coalescent model (MSCM) (Pamilo and Nei, 1988), is the most prevalent and has so far received most attention (Edwards, 2009; Davidson et al., 2015). High levels of ILS are most likely to occur when there is a short time between speciation events, i.e., when internal branches of the species tree are short (Maddison, 1997; Whitfield and Lockhart, 2007). The concatenation approach combines the information from all available alignments into a single alignment and can mitigate low phylogenetic signal-to-noise problems (Philippe et al., 2005; de Queiroz and Gatesy, 2007). However, it ignores ILS and may, conversely to coalescence-based approaches, return highly supported but erroneous estimates of relationships in or near the anomaly zone, a region of tree space caused by successive rapid speciation events in the species tree, in which the most probable gene tree topology differs from the species tree topology (Degnan and Rosenberg, 2006; Kubatko and Degnan, 2007; Liu and Edwards, 2009; Edwards et al., 2016; Mendes and Hahn, 2018).

Despite this drawback concatenation often performs well under many conditions, even in the presence of moderately high ILS levels (Bayzid and Warnow, 2013; Mirarab et al., 2016). Unlike concatenation, ASTRAL and several other coalescence-based methods can accommodate gene tree discordance due to ILS, and are statistically consistent under the MSCM (Mirarab and Warnow, 2015; Roch and Steel, 2015). Yet, coalescence-based approaches have been criticized for violating the MSCM assumptions such as error-free gene trees, absence of recombination within genes and free recombination between genes (Gatesy and Springer, 2014). While violations of the assumption of free recombination between loci can result in inaccurate phylogenetic estimates (Wang and Liu, 2016), both simulation and empirical studies have indicated that analyses using ASTRAL are largely robust to inclusion of recombinant loci (Lanier and Knowles, 2012; Wang and Liu, 2016; Folk et al., 2017; Morales-Briones et al., 2018). Nevertheless, coalescent-based methods can be sensitive to gene tree error, which can be alleviated using more informative genes and/or collapsing poorly supported relationships in gene trees prior to species tree inference (Zhang et al., 2018).

In Rubioideae, concatenation- and coalescence-based approaches generated highly similar topologies. However, one notable and highly supported topological conflict between the two approaches was detected: in the concatenated tree Ophiorrhizeae and Colletoecemateae + Seychelleeae formed a clade (BS = 100; Figure 2), whereas Ophiorrhizeae and Urophylleae s.s. formed a clade (LPP = 0.99; Figure 1) in the coalescence tree. This part of the tree has successive relatively short internal branches, a typical pattern of the anomaly zone, and indicate that the divergent placements of Ophiorrhizeae can be due to ILS and how it is differently accounted for in the two analytical approaches (Linkem et al., 2016). While inaccurate ortholog inference as well as gene tree error can generate gene tree incongruence, the pattern of gene tree quartet frequencies (Figure 1) with one main topology and balanced frequencies among the alternative topologies is more compatible with ILS as the main source of incongruence (Zou et al., 2008; Degnan and Rosenberg, 2009). It should be noted that the same incongruence is found also between the two analyses of the paralog-filtered dataset (Supplementary Figures 3, 4), but the support for the Ophiorrhizeae + Urophylleae s.s. branch was low (LPP = 0.91) and a polytomy could not be rejected. In contrast, this incongruence was not observed in the trees resulting from the analyses of the two CDS datasets, but except for the well-supported Colletoecemateae + Seychelleeae branch, all other intertribal relationships within the SCOUT clade were poorly supported in those trees and polytomies could not be rejected (Supplementary Figures 1, 2, 5, 6).

### Phylogeny of Rubioideae

#### SCOUT clade

Studies addressing the deepest divergences in Rubioideae have often come to different conclusions but have most commonly involved the relative placements of the tropical African tribe Colletoecemateae, the Australasian tribe Ophiorrhizeae and the pantropical tribe Urophylleae. Analyses based on chloroplast sequence data have shown contradictory results; early studies based on Sanger sequencing of a few selected markers often found Colletoecemateae as sister to the remaining members of the subfamily (Robbrecht and Manen, 2006; Rydin et al., 2008, 2009a) but using a relaxed-clock model, Wikström et al. (2015) instead found Urophylleae as sister to the remaining tribes. More recent phylogenomic work has also resulted in topological incongruence; Ophiorrhizeae was sister to the remaining Rubioideae based on plastome data (Wikström et al., 2020), whereas the study by Rydin et al. (2017) based on mitochondrial data instead found Colletoecemateae + Ophiorrhizeae as sister to the remaining subfamily. The few analyses using nuclear data have consistently found a Colletoecemateae-Ophiorrhizeae-Urophylleae clade as the sister-group to the remaining Rubioideae, a result first reported by Rydin et al. (2009a) based on nrITS data and more recently also found based on nuclear ribosomal cistron (Wikström et al., 2020) and Angiosperm353 data (Antonelli et al., 2021). Based on plastid markers, the monogeneric seychellean tribe Seychelleeae was recently found to be sister-taxon to the species-poor monogeneric tropical African tribe Colletoecemateae (Razafimandimbison et al., 2020), a relationship that is confirmed here. Our analyses consistently resolved a Seychelleeae-Colletoecemateae-Ophiorrhizeae-Urophylleae s.s. clade as the sister to the remaining subfamily Rubioideae, and we further show that the African genus *Temnopteryx* belongs in this clade, the SCOUT clade. Early classifications have differed in the tribal and subfamilial position of *Temnopteryx*, summarized by Khan et al. (2008), Smedmark et al. (2008). Khan et al. (2008) was the first phylogenetic study based on molecular to include *Temnopteryx*, and they showed that it belongs to Rubioideae although they did not resolve its position within the subfamily. In subsequent work based on molecular data (Smedmark et al., 2008, 2010; Smedmark and Bremer, 2011; Yang et al., 2016), *Temnopteryx* has been resolved as sister to the (remaining) tribe Urophylleae, although not always with high support. Here we instead find *Temnopteryx* strongly supported as sister to the remaining members of the SCOUT-clade (Figures 1, 2).

#### Lasiantheae-Perameae

The second deepest split in the Rubioideae phylogeny separates a Lasiantheae-Perameae clade from the remaining members of the subfamily, i.e., a clade comprising the Psychotrieae and Spermacoceae alliances and the tribe Coussareeae. The sister-group relationship between the monogeneric tribe Perameae and Lasiantheae was first found by Andersson and Rova (1999) based on plastid *rps16* intron data and was considered surprising at the time, as there is no obvious morphological similarity between the two tribes. Although the tribe Perameae has not been as frequently sampled as Lasiantheae in molecular phylogenetic studies of Rubiaceae, the Lasiantheae-Perameae clade is well founded based on DNA sequence data with several subsequent studies supporting this relationship (e.g., Piesschaert et al., 2000; Smedmark et al., 2014; Antonelli et al., 2021; this study). While Perameae are tiny herbaceous plants with dry capsular fruits, Lasiantheae are woody, shrubby plants with fleshy drupes (Bremer and Manen, 2000; Smedmark et al., 2014). A feature they have in common is a solitary ovule in each locule, but the feature is found in several other members of Rubioideae as well (Bremer and Manen, 2000; Smedmark et al., 2014). The two tribes are thus morphologically distinct and we agree with previous authors (Andersson and Rova, 1999; Bremer and Manen, 2000) that merging the two tribes into Perameae should be avoided as it would create a morphologically undefinable taxon.

#### Coussareeae-Spermacoceae alliance

A notable result from the study by Wikström et al. (2020) was the placement of the tribe Coussareeae as sister to the Spermacoceae alliance on the basis of nuclear ribosomal data. The result conflicted with their own as well as previous results based on plastid data (Rydin et al., 2008, 2009a; Wikström et al., 2015, 2020; Neupane et al., 2017), plastid data + nrITS (Rydin et al., 2009b) and mitochondrial data (Rydin et al., 2017), which have all consistently supported the Coussareeae as sister to a clade comprised by the Spermacoceae and the Psychotrieae alliances. Our work (based on nuclear data) is congruent with the analyses of nuclear ribosomal cistron data by Wikström et al. (2020) regarding the relative positions of these three groups, but while the support for the sister relationship between the Coussareeae and the Spermacoceae alliance is high in the concatenated tree (BS = 100, Figure 2) it is relatively low in the coalescence-based tree (LPP = 0.75, Figure 1). The branch uniting Coussareeae and Spermacoceae alliance is short and gene tree heterogeneity high with quartet frequencies fairly even. Taken together, these findings indicate that ILS is the probable explanation for observed gene tree heterogeneity, and that a rapid speciation event may constitute the origin of these two sister clades.

#### Psychotrieae alliance

The nuclear phylogeny presented here includes representatives of all nine currently recognized tribes of the Psychotrieae alliance (Razafimandimbison et al., 2008, 2017) and shows, in contrast to previous studies based on Sanger-data, strong support across almost all relationships (including all inter-tribal relationships). Our study further supports the rare case of an evolutionary change from one-seeded carpels to many-seeded carpels found in the Psychotrieae alliance (Razafimandimbison et al., 2008), with Schradereae being the sole tribe with numerous ovules per locule. Our results are congruent with previously published phylogenies based on nuclear and mitochondrial data, although the studies by Rydin et al. (2017) and Wikström et al. (2020) did not include Schradereae and Antonelli et al. (2021) did not include Schradereae and Mitchelleae. Schradereae is here resolved as sister to the clade containing Prismatomerideae and the Morindeae-Mitchelleae clade. This clade is in turn sister to a clade comprising Gaertnereae and the Palicoureeae-Psychotrieae clade. The positions of the monogeneric African tribes Schizocoleeae and Craterispermeae as successive sisters to all other members of Psychotrieae alliance is consistent also with previous analyses based on plastid data. However, analyses of plastid data have found a sister-relationship between Gaertnereae and Prismatomerideae together sister to the Morindeae-Mitchelleae clade (Wikström et al., 2020; Antonelli et al., 2021), or Gaertnereae forming a clade with Schradereae, Morindeae and Mitchelleae together sister to the Palicoureeae-Psychotrieae clade with Prismatomerideae placed as sister to those two clades (Wikström et al., 2015).

Analyses based on combined plastid and nuclear ribosomal markers have largely produced results consistent with our results but have supported a Craterispermeae + Prismatomerideae clade (Razafimandimbison et al., 2008), or the placement of Gaertnereae in a clade together with Schradereae, Prismatomerideae, Mitchelleae and Morindeae (Razafimandimbison et al., 2017). It is interesting to note that the nuclear results and mitochondrial results agree and are both in conflict with the plastid signal. Such discrepancies between results obtained with nuclear and mitochondrial data on one hand and plastid data on the other may be the result of old introgression events. However, the relatively short branch lengths and the quartet frequencies along the backbone nodes of the Psychotrieae alliance indicate relatively high levels of ILS during the early diversification of this clade.

#### Spermacoceae alliance

Resolving relationships in Spermacoceae alliance has been problematic, with relationships either unconvincingly supported or showing discordant topologies. In the Spermacoceae alliance our results support the position of the Danaideae-Knoxieae-Spermacoceae clade as sister taxon to the remaining members of the alliance. Several previous studies have shown results congruent with this, including a study based on mitochondrial data Rydin et al. (2017), the plastome-based phylogenomic analyses in Wikström et al. (2020), and analyses of a few selected plastid markers alone or in combination with nuclear ribosomal ITS (nrITS, e.g., Rydin et al., 2009a; Krüger, 2014; Wikström et al., 2015; Thureborn et al., 2019). Other analyses based on a few selected plastid markers, alone or in combination with nuclear ribosomal ITS, have not produced results congruent with ours, but have often found Danaideae as sole sister to the remaining members of the alliance (e.g., Bremer and Manen, 2000; Bremer and Eriksson, 2009; Rydin et al., 2009b; Yang et al., 2016). Analyses of the nuclear ribosomal cistron recovered yet another unexpected relationship with Anthospermeae sister to the Knoxieae-Spermacoceae clade, and Danaideae nested in a clade comprising the other sampled members of the alliance (Wikström et al., 2020). Further, the results presented by Antonelli et al. (2021) based on nuclear Angiosperms353 data showed surprisingly Argostemmateae (represented by one sample, *Mycetia* sp.) followed by Spermacoceae (represented by one sample, *Spermacoce* sp.) as successive sisters to the rest of Spermacoceae alliance. Those same samples were included in the present study (Figures 1, 2), yielding other (more expected) topological placements of these samples. The discordance between our results and those of Antonelli et al. (2021) regarding the phylogenetic placement of these two samples may potentially be explained by the denser taxon sampling in the present study, for example in terms of tribes (11 vs. 8) and genera (40 vs. 10).

In the Rubieae complex, our results support the sister-relationship between Theligoneae and Putorieae and corroborate previous results based on nuclear ribosomal cistron Wikström et al. (2020) and Angiosperm353 data (Antonelli et al., 2021). Previous studies utilizing plastid data or a combination of plastid and nrITS data have either shown results consistent with our result (Yang et al., 2016; Antonelli et al., 2021; Rincón-Barrado et al., 2021) or have instead resolved Theligoneae and Rubieae as sister groups (e.g., Backlund et al., 2007; Bremer and Eriksson, 2009; Rydin et al., 2009b; Deng et al., 2017; Ehrendorfer et al., 2018; Wikström et al., 2020), a result also found when analyzing mitochondrial data (Rydin et al., 2017). While obvious morphological similarities supporting the Theligoneae + Rubieae clade seem to be lacking (Ehrendorfer et al., 2018) there are some morphological characters shared between some Putorieae species and members of clades within Rubieae (Natali et al., 1995; Ehrendorfer et al., 2018). Interestingly a recent study (Bordbar et al., 2021) found on the basis of the plastid *trnL-F* marker that *Plocama rosea* (Hemsl. ex Aitch.) M.Backlund and Thulin (= *Aitchisonia rosea* Hemsl. ex Aitch.) formed a clade with Rubieae, with Theligoneae and a clade containing the remaining sampled Putorieae/*Plocama* species as successive sisters to this clade. Based on those results the authors resurrected the monospecific genus *Aitchisonia* Hemsl. ex Aitch., and described the new monogeneric tribe Aitchisonieae to accommodate *A. rosea*. However, based on nrITS data the placement of *Plocama rosea* was inconclusive (Bordbar et al., 2021).

The sister group to the Rubieae complex is in our trees the tribe Paederieae, a relationship previously found in analyses based on nuclear and/or plastid data (Robbrecht and Manen, 2006; Rydin et al., 2009a,b; Wikström et al., 2015, 2020; Yang et al., 2016; Antonelli et al., 2021), although based on data from the mitochondrion this relationship was intervened by Argostemmateae (Rydin et al., 2017).

In our trees Anthospermeae, the Dunnieae + Cyanoneuroneae clade and Argostemmateae are supported as sequential sister groups to the Paederieae-Rubieae complex clade, a result fully congruent with the analyses of plastid data in Wikström et al. (2020). Other previous studies using plastid data and a combination of plastid and nuclear nrITS data have often been partly congruent with our results. The Anthospermeae-sister relationship has often been well supported but relationships among representatives of the remaining groups have generally been poorly supported (Rydin et al., 2009a,b; Wikström et al., 2015; Yang et al., 2016). Analyses of mitochondrial data have instead found Anthospermeae + Dunnieae, Paederieae and Argostemmateae as successive sisters to the Rubieae complex (Rydin et al., 2017). Analyses of the nuclear ribosomal cistron supported Anthospermeae as sister to the Knoxieae-Spermacoceae clade, and Danaideae nested in a clade containing the other sampled members of the alliance (Wikström et al., 2020). Previous analyses utilizing nuclear Angiosperm353 data (Antonelli et al., 2021) found Argostemmateae placed as sister to the remaining Spermacoceae alliance (represented by Spermacoceae, Cyanoneuroneae, Anthospermeae, Paederieae and the Rubieae complex) in their coalescence-based tree (their concatenation-based tree was inconclusive except for the Paederieae-Rubieae complex phylogeny). However, our respective results are not fully comparable since Argostemmateae in our study includes also the single representative sample of Argostemmateae (*Mycetia* sp.) used in Antonelli et al. (2021) and the conflicting signal may thus be due to low sampling in their study relative to ours.

Our results support the close relationship between the two relatively recently described monogeneric tribes Dunnieae (China) (Rydin et al., 2009b) and Cyanoneuroneae (Borneo and Sulawesi) (Ginter et al., 2015). This result is congruent with Ginter et al. (2015) who, based on combined plastid and nuclear (nrETS and nrITS) data, found that those two tribes formed a clade that also included yet another recently described monogeneric tribe, the Foonchewieae from China (Wen and Wang, 2012). Thureborn et al. (2019) included representation from all these three tribes and found, based on plastid data, that they form a clade together with Argostemmateae (appendix B in Thureborn et al., 2019). Recent studies addressing major relationships in Rubiaceae have otherwise typically only included representation from one of these three tribes [for example, Antonelli et al. (2021) included Cyanoneuroneae and Rydin et al. (2017) and Wikström et al. (2020) included Dunnieae], but the close relationship between Foonchewieae and Dunnieae has been confirmed in several studies based on analyses of plastid data (Wikström et al., 2015; Yang et al., 2016). However, a highly unexpected placement of Cyanoneuroneae was found in the plastid tree of Antonelli et al. (2021); the Spermacoceae alliance excluding Cyanoneuroneae was strongly supported and Cyanoneuroneae was with strong support deeply nested in a clade comprising the sampled members of the Psychotrieae alliance. This result is not retrieved in other previous studies, nor in the results of the present study.

#### Infratribal relationships

Within tribes, our results reveal novel relationships and place a genus previously not included in phylogenetic analyses based on molecular data. Here we discuss intergeneric relationships within tribes whenever relevant and/or possible considering our sample of taxa.

##### Ophiorrhizeae

Within the Ophiorrhizeae, *Neurocalyx* is sister to the remaining tribe, and *Kajewskiella* is sister to *Lerchea* + *Ophiorrhiza* (Figures 1, 2). The results are consistent with those of a recent study that investigated the phylogeny of Ophiorrhizeae using extensive species representation, five molecular markers and morphological considerations (Razafimandimbison and Rydin, 2019). Material for DNA-sequencing of *Kajewskiella* was unavailable to the authors at the time, but they predicted its inclusion in Ophiorrhizeae based on morphology, presumably sister to *Xanthophytum* (Razafimandimbison and Rydin, 2019). A later study included molecular data from *Kajewskiella* and confirmed its phylogenetic position in Ophiorrhizeae (Antonelli et al., 2021), although limited taxon sampling prevented further conclusions. The exact position of *Kajewskiella* within Ophiorrhizeae remains unresolved. The affinity to *Xanthophytum* was first suggested by Tange (1995) who discovered raphides in bract tissue in the inflorescences, “…*indistinguishable from those found in Xanthophytum*” (citation from Tange, 1995). The author found additional morphological indications of an affinity to *Xanthophytum* (Tange, 1995), and this was thus endorsed in the recent (greatly expanded) study of Ophiorrhizeae by some of us (Razafimandimbison and Rydin, 2019). Furthermore, Tange (1995) added information on *Kajewskiella* to Axelius’s (1990) morphological data matrix of *Xanthophytum*, and reported that his parsimony analysis of the data placed *Kajewskiella* with *Xanthophytum papuanum, X. grandiporum, X. magnisepalum*, and *X. nitens*, a clade that had a derived position in Axelius’s work (Axelius, 1990). There is thus ample morphological support for the reduction of *Kajewskiella* into *Xanthophytum*, as suggested by Tange (1995), but the hypothesis remains to be tested using molecular data from an adequate sample of species within the entire tribe, analyzed with state-of-the-art analytical tools.

##### Schradereae

In the tribe Schradereae, the Southeast Asian genus *Lecananthus* (Puff et al., 1998a) was recently shown to be nested in *Schradera* (Razafimandimbison et al., 2017), a result corroborated in the current study and further confirming the paraphyly of *Schradera* as delimited by Puff et al. (1998b). However, here *Lecananthus* is more closely related to the Asian species *Schradera nervulosa* than to the neotropical species *Schradera rotundata*.

##### Anthospermeae

We included representatives from 11 of the 12 genera of the Anthospermeae; only *Nenax* was not sampled since a recent study showed that species of *Nenax* are intermixed with those of *Anthospermum* in an *Anthospermum-Nenax* clade (Thureborn et al., 2019). Our results support the position of the South African genus *Carpacoce* as sister to a clade that unites an African clade and a Pacific clade, which is entirely congruent with results in Thureborn et al. (2019). Within the African clade, the positions of the southeastern Africa-centered genus *Galopina* and the Macaronesian genus *Phyllis* and their relationship(s) to *Anthospermum-Nenax* have been problematic with incongruent results and poor statistic support (Anderson et al., 2001; Yang et al., 2016; Thureborn et al., 2019). Here, *Galopina* and *Phyllis* form a highly supported clade (Figures 1, 2), a relationship that has been suggested based on morphology (Sunding, 1979). It is worth noting that although this sister relationship is highly supported in all concatenated trees, only the supercontig dataset that includes non-coding data had the power to reject the null hypothesis of a polytomy for this relatively short branch. The quartet frequencies (Figure 1) indicate that ILS contributes to a large proportion of the gene tree incongruence, which in combination with a relatively short branch suggest rapid speciation in the diversification history of this group. Within the Pacific clade, our results support the Australian genus *Durringtonia* as sister to the remaining clade, which in turn comprises (a) *Leptostigma* and *Pomax* + *Opercularia*, and (b) *Normandia* and *Coprosma* + *Nertera*. The analyses of nuclear data by Thureborn et al. (2019) placed *Durringtonia* in the latter clade but results were otherwise completely congruent with those presented here. Our results show that the subtribal classification of Anthospermeae, based mainly on flower and fruit characters (Puff, 1982), needs revision. The Australian subtribe Operculariinae (*Pomax* and *Opercularia*) is monophyletic but is nested in the paraphyletic subtribe Coprosminae. Analyses of plastid data have previously indicated that both these subtribes are non-monophyletic (Thureborn et al., 2019) but support values were not significant. It should further be noted that Thureborn et al. (2019) detected some cases of supported cytonuclear discordance in the tribe. Generic interrelationships in Anthospermeae should be further investigated using genomic data.

##### Argostemmateae

Five of the genera we included in the present study were resolved in the Argostemmateae: *Argostemma, Clarkella, Neohymenopogon, Mouretia*, and *Mycetia. Argostemma* is sister to the remaining tribe. *Clarkella*, a small Asian herbaceous genus containing a single species (*Clarkella nana*), is here addressed for the first time using molecular data (but see Figure 2C in Yang et al., 2016), and the results show that it belongs in Argostemmateae, sister to *Neohymenopogon* + a *Mycetia–Mouretia* clade (Figures 1, 2). *Clarkella* is currently placed in its own tribe Clarkelleae (Deb, 2001), but it was placed in Argostemmateae in earlier classifications (Verdcourt, 1958; Bremekamp, 1966). It was later excluded from Argostemmateae based on flower and pollen characters (Bremer, 1987) but both vegetative and fruit characters of *Clarkella* resemble those of some species of *Argostemma* (Puff and Chayamarit, 2008).

The intergeneric relationships within Argostemmateae are identical between the two inference methods we used (Figures 1, 2) and all but one node (the *Neohymenopogon* + *Mycetia*–*Mouretia* clade in the coalescent tree, where a polytomy could not be rejected; Figure 1) are strongly supported. Our results differ, however, from those in previous studies (which are based on limited amount of molecular data, i.e., Rydin et al., 2009b; Ginter et al., 2015; Yang et al., 2016). Results in those studies are not always well supported and we too find indications of inconsistency regarding relationships in Argostemmateae. For example, in the analyses of the full CDS data, the coalescent tree supports a *Neohymenopogon* + *Mouretia* clade (Supplementary Figure 1), and the concatenation tree was inconclusive (i.e., support values were below 95%) for several relationships (Supplementary Figure 2) and inconsistent with the coalescent tree. Addition of data in the form of genes or longer sequences has been shown to lead to more congruence between species tree estimates (Cai et al., 2021; Gardner et al., 2021), and such a trend seems to be present also in Argostemmateae. Relationships in the tribe should nevertheless be investigated further, preferably also including the Asian and herbaceous genus *Leptomischus*, which recently was proposed to be sister to the remaining Argostemmateae based on plastid (*rbcL*) data (Razafimandimbison and Rydin, 2019).

##### Paederieae

We included five (*Leptodermis*, *Paederia*, *Pseudopyxis*, *Serissa*, and *Spermadictyon)* of the six currently recognized genera of Paederieae (Backlund et al., 2007; Rydin et al., 2009b). One of those is *Pseudopyxis* (*P. heterophylleae*), a genus here included in a molecular study for the first time. Its inclusion in the Paederieae is in line with Puff’s (1982) classification of this tribe on the basis of morphology and geography. *Pseudopyxis* (three species) comprises perennial herbs occurring in China and Japan, and is here sister to a mainly woody Southeast Asian clade consisting of *Spermadictyon, Leptodermis*, and *Serissa.* Sister to those four genera is *Paederia*, a genus of pantropical and woody climbers. Our results agree well with the informal infratribal groupings suggested by Puff (1989) based on morphology and geography and are also consistent with previous molecular results based on plastid data (Backlund et al., 2007; Yang et al., 2016) as well as results based on a combination of plastid data and nrITS data (Rydin et al., 2009b). The Southeast Asian genus *Saprosma* is unfortunately not represented in our study. The genus was placed in Paederieae by Robbrecht (1993) based on morphology, and most subsequent work based on molecular data has since supported this, placing *Saprosma* either as sister to all other members of Paederieae (Rydin et al., 2009b) or sister to *Paederia* (Yang et al., 2016). It was however sister to the Rubieae complex in Backlund et al. (2007).

## Data availability statement

The raw data generated for the present study are deposited in the European Nucleotide Archive (ENA) under study accession number PRJEB53647. The ENA sample accession numbers of all the samples are available in Supplementary Table 1. The target file used for HybPiper assembly and the assembled sequences are uploaded to Dryad Digital Repository, 10.5061/dryad.d7wm37q44.

## Author contributions

OT carried out the molecular experiments, post-sequencing bioinformatics analyses, and the phylogenetic analyses, and wrote the manuscript with input from all authors. All authors contributed to conception and design of the study, read, and approved the submitted version.

## Footnotes

1
http://www.arborbiosci.com/mybaits-manual
