# Supplementary material for: Target capture data resolve recalcitrant relationships in the coffee family (Rubioideae, Rubiaceae)
Source: Front Plant Sci. 2022 Sep 8;13:967456. doi: 10.3389/fpls.2022.967456 (PMC9493367; doi:10.3389/fpls.2022.967456)
Supplement: Supplementary file 6 [file Image_3.pdf]

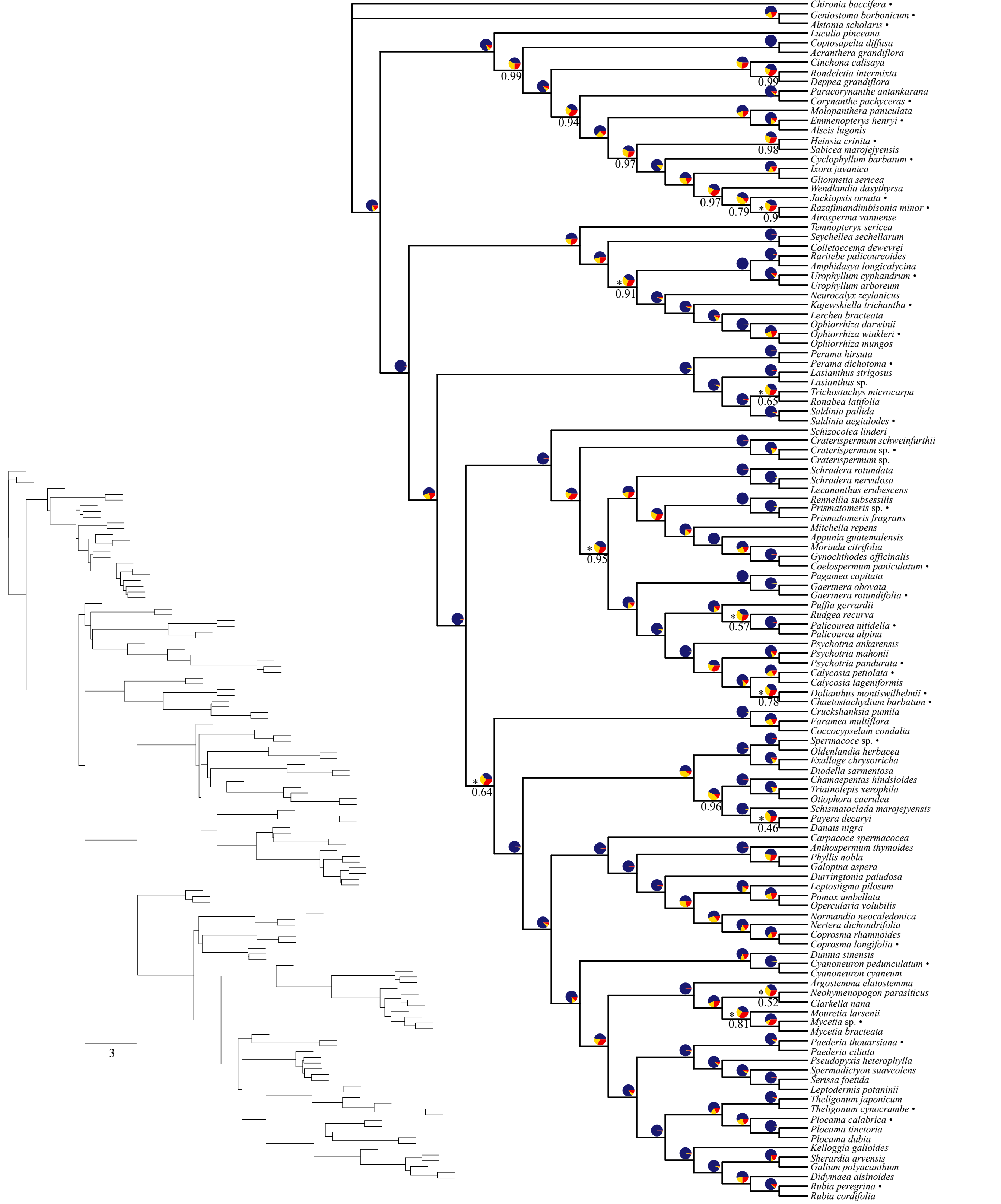

Supplementary Figure 3. Coalescent-based species tree estimated using ASTRAL on the paralog-filtered supercontig dataset. Numbers below branches denote local posterior probability (LPP) support values. Only support values smaller than 100% are shown. Pie charts show relative frequencies of the three quartet topologies around the branch (blue = congruent with species tree, yellow = first alternative topology, red = second alternative topology). Asterisks next to pie charts indicate failure to reject the hypothesis that the branch is a polytomy. Bullets after species names indicate samples downloaded from ENA. Inset shows branch lengths in coalescent units.
