# Supplementary material for: Target capture data resolve recalcitrant relationships in the coffee family (Rubioideae, Rubiaceae)
Source: Front Plant Sci. 2022 Sep 8;13:967456. doi: 10.3389/fpls.2022.967456 (PMC9493367; doi:10.3389/fpls.2022.967456)
Supplement: Supplementary file 7 [file Image_4.pdf]

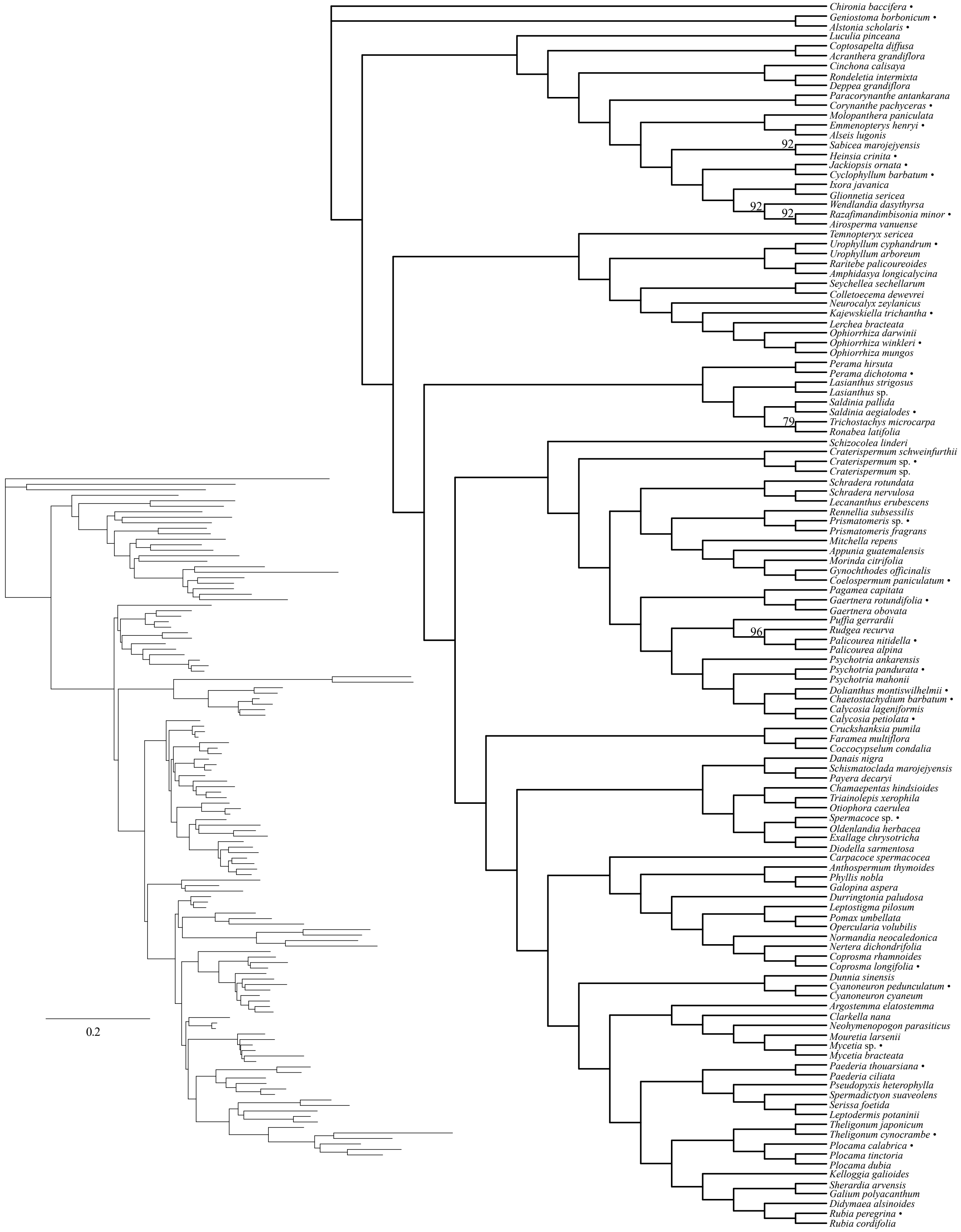

Supplementary Figure 4. Concatenation-based tree estimated using IQ-TREE on the paralog-filtered supercontig dataset. Numbers above branches denote ultrafast bootstrap (BS) support values. Only support values smaller than 100% are shown. Bullets after species names indicate samples downloaded from ENA. Inset shows branch lengths in number of substitutions per site.
